# Equity Theory Ratios as Causal Schemas

**DOI:** 10.3389/fpsyg.2016.01257

**Published:** 2016-08-19

**Authors:** Alexios Arvanitis, Alexandra Hantzi

**Affiliations:** ^1^Department of Psychology, University of CreteRethymnon, Greece; ^2^Department of Psychology, Panteion UniversityAthens, Greece

**Keywords:** social exchange, justice, equity, communicative rationality, psychological contract, attribution

## Abstract

Equity theory approaches justice evaluations based on ratios of exchange inputs to exchange outcomes. Situations are evaluated as just if ratios are equal and unjust if unequal. We suggest that equity ratios serve a more fundamental cognitive function than the evaluation of justice. More particularly, we propose that they serve as causal schemas for exchange outcomes, that is, they assist in determining whether certain outcomes are caused by inputs of other people in the context of an exchange process. Equality or inequality of ratios in this sense points to an exchange process. Indeed, Study 1 shows that different exchange situations, such as disproportional or balanced proportional situations, create perceptions of give-and-take on the basis of equity ratios. Study 2 shows that perceptions of justice are based more on communicatively accepted rules of interaction than equity-based evaluations, thereby offering a distinction between an attribution and an evaluation cognitive process for exchange outcomes.

## Introduction

Equity theory was introduced in the study of organizational settings to examine justice in the workplace environment (Adams, 1963), but was soon formulated as a general theory of interpersonal relationships (Walster et al., 1973) that treats social interaction as an exchange of resources among actors. It is part of a broader set of social exchange theories, which have been extensively applied in understanding workplace behavior, although their concepts are not strictly defined and articulated (Cropanzano and Mitchell, 2005).

Equity theory specifically argues that resources should be used in a way so that inputs and outcomes of an exchange process are proportional. In this sense, *equity ratios* (which can be perceived as ratios of outcomes to inputs or vice versa) should be equal. The main reasoning behind this is that *rational* people would accept no other arrangement in their effort to maximize outcomes and manage their resources effectively (see Walster et al., 1973). In fact, equity theory is strongly based on the *instrumental rationality* postulate and essentially argues that inputs and outcomes are the main determinants of the evaluation process of an exchange as just or unjust.

It is not necessary, though, to view rationality only in terms of the effectiveness of goal-directed actions and, therefore, the effective use of exchange participants’ resources; rationality can be treated more broadly in terms of the ability of exchange participants to reach a common understanding about their shared ‘lifeworld’ (see Habermas, 1985). In short, whatever brings about an agreement that is communicatively achieved can serve as a criterion for the evaluation of an exchange situation.

In this sense, equity ratios may indeed include necessary elements of any exchange process –inputs and outcomes– but they do not have a hidden property of ‘compulsory’ equality. In fact, they can be treated as cognitive elements of a causal schema that processes how exchange outcomes are linked to exchange inputs. This causal schema could be derived from an implicit knowledge about the structure of the world, or more specifically about the nature of social interaction (Kelley, 1973).

Our purpose is to examine whether equity ratios in essence illustrate a relationship between cause and effect which is necessary to the cognitive process of the attribution of exchange outcomes, rather than to the cognitive process of the evaluation of the exchange; the latter process would serve the purpose of establishing the appropriate norm to the effected exchange and would often go beyond equity considerations. We will further examine, consistent with the *communicative rationality* view of Habermas (1985), whether justice is evaluated in terms of the rules that can elicit agreement of exchange participants.

### Equity Ratios and Attribution of Outcomes

Attribution theory is concerned with the way in which people answer causal questions, i.e., questions beginning with ‘why.’ Originating in Heider’s conception of people as causal ‘origins’ (Heider, 1944), attribution theory has offered valuable insight into how people are able to attribute actions to internal dispositions of actors (Jones and Davis, 1965) or derive their own attitudes from observing their own actions (Bem, 1967). Research in this field has remained diverse and its boundaries have never really been formalized (Weiner, 2008). Few in this field, however, would argue against Heider (1958) who treated people as naive scientists that adopt principles to produce cause-effect relationships in a similar manner that a scientific system is formulated. At the same time it is accepted that on a more practical level, people often employ causal schemas, that is, abstract ideas about the nature of causal factors, since they lack the motivation, the time, or the knowledge to make proper scientific evaluations (Kelley, 1973).

Our research is not the first to examine equity and attribution with regard to exchange outcomes. Hegtvedt et al. (1993) studied the ways in which perceived equity was associated with the attribution of outcomes to the self or the other. They found that the greater the perception of equity, the greater the attribution of outcomes to the self. However, they did not propose a cognitive mechanism for how equity is linked to the attributions, or when people view such a situation as an exchange. To our knowledge, there has been no explicit reference regarding if and how equity ratios can be considered causal schemas for exchange outcomes.

In a two–party interaction between A and B, one way the equity postulate can be portrayed is the following:

$$\frac{Outcomes\,\;A}{Inputs\,\;A}=\frac{Outcomes\,\;B}{Inputs\,\;B}$$

**FIGURE 1:**
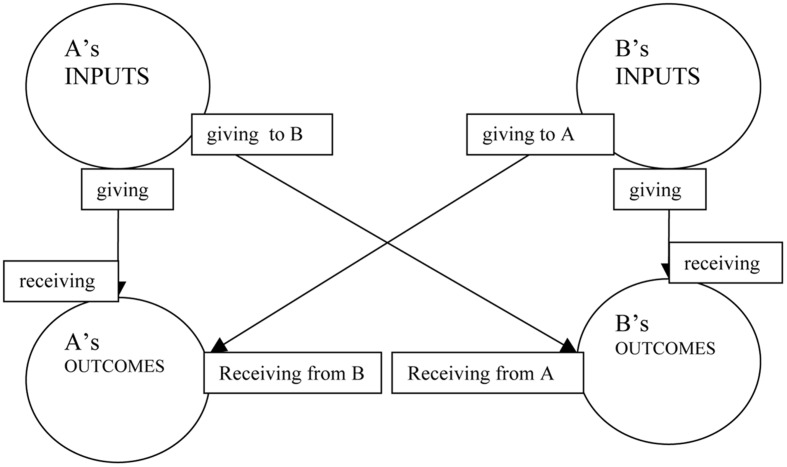
**The attribution of exchange outcomes in a two-party interaction**.

One of the problems that have been identified in equity theory is that inputs and outcomes are often difficult to distinguish empirically (Cook and Parcel, 1977). Our proposal is to treat equity theory ratios as causal schemas where inputs are causes and outcomes are effects. A’s and B’s actions (inputs) can influence both actors’ outcomes (see **Figure 1**, where causal relationships are illustrated by the use of arrows). If there is no other factor (such as other actors, chance etc.) that influences the actors’ outcomes, any disproportionality in inputs and outcomes of one actor first of all implies that one actor has influenced the other. If we limit our analysis to positive influence, i.e., the use of positive resources, an unfavorable disproportionality to A (A’s outcomes/A’s inputs < B’s outcomes/B’s inputs) means that A has used resources in favor of B’s outcomes. In this sense A has ‘given,’ B has ‘received’ and an exchange has taken place. Perceptions of ‘receiving’ essentially describe a cause-effect relationship between one’s outcome and another person’s input and are the result of the attribution process of one’s outcome to another person’s input.

The above reasoning may be clarified through an example. Let us for a moment assume that there is no exchange and A performs a simple goal-directed action: She is making pancakes. Inputs include her effort, the cost of the ingredients, and the use of the oven, while the outcome includes the quality and quantity of the pancakes that A consumes. Without B, outcomes can only be attributed to A’s inputs (illustrated by the arrow from A’s inputs to A’s outcomes, in **Figure 1**). Now, if B comes into the picture, he may eat part of the pancakes without offering any input or very little. This is an example of a disproportional situation, where B receives from A (illustrated by the arrow from A’s inputs to B’s outcomes), since the pancakes he eats are attributed to A’s inputs. The less inputs B provides and the more outcomes he reaps, the greatest the disproportionality and the perception of B’s receiving. On the other hand, B can sit in the same kitchen, make his own pancakes and consume only the pancakes he made (illustrated by the arrow from B’s inputs to B’s outcomes). In this case, the outcomes of A can only be attributed to the inputs of A and the outcomes of B to the inputs of B. This would be a proportional situation where no real exchange has taken place. B can also share his ingredients with A, collaborate with A and in the end share the pancakes in a proportional manner. From the bulk of pancakes that both A and B made, we can attribute the existence of some pancakes to A and some to B, depending on the amount of their inputs, and deduce who has received from the other or, in general, whether an exchange has taken place.

**FIGURE 2:**
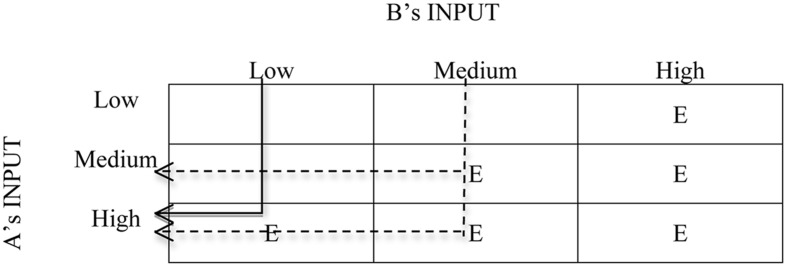
**Causal schema for compensatory causes in the form of Kelley (1973).** (E = high outcomes for A).

The causal schema for a high outcome for A may be depicted in the more familiar form of a compensatory cause schema (Kelley, 1972, 1973), where two quantitatively graded causes contribute to the effect (**Figure 2**). The inputs of A and B contribute to the final high outcome of A. However, only when the input of B is low can we deduce that the cause of the high outcome for A is A’s high input (indicated by the solid arrow). In the cases of high or medium input of B, one cannot easily infer the extent that the input of A contributed to the final high outcome (indicated by the dotted arrows). Unless, that is, one knows more about the outcome of B. The case of exchange is too complicated to be depicted in a two-dimensional causal schema because it is not only the inputs that are taken into account, but also the outcomes of both parties. A straightforward way to portray this type of schema is to resort to **Figure 1** or the equity ratios themselves.

It is also important to note that the current analysis is presented with slight but important differences compared to mainstream attribution analyses. Most psychological research is based on the dichotomy between internal and external attribution (Hewstone, 1989), often failing to distinguish among the different social objects: task, self, other, social unit (Lawler, 2001). The social unit can be an exchange relationship, a network, or group. Indeed an exchange relationship may offer an attractive explanation for an outcome (Eberly et al., 2011). Understanding how and when people attribute outcomes to the other person in the context of an exchange relationship offers a more fine-grained explanation than the one offered by the usual internal-external dichotomy. Our reasoning and measures concerning attribution have been developed mostly to reflect an inter-individual or dyadic level of analysis (Krasikova and LeBreton, 2012; Tse and Ashkanasy, 2015) than the usual intra-individual level of analysis (for more about levels of analyses in social psychology see Doise, 1980).

On a dyadic level, interactions are often assumed to be exchanges of resources such as love, information, or money (Foa and Foa, 1974) but the case in which people actually treat interactions as exchanges is rarely examined. Our proposed approach rests on a fundamental assumption: *People can attribute outcomes to actions and to resources of their own and/or those of other people.* When they perceive that it is not only their own actions or their own resources (their own inputs) that influence their outcomes, they partly attribute their outcomes to the inputs of other people. In this case, they perceive that they ‘receive.’ The ratios of equity theory implicitly point to an attribution process, and yet have not been used and tested in such a way explicitly. Proportionality or disproportionality (i.e., equal or unequal ratios) may contribute to our understanding of how individuals exchange resources once we take into account the attribution process concerning resources and outcomes.

*Should* there be proportionality though (i.e., is proportionality an imperative) as equity theory argues? The ‘ought’ element is not an inherent property of equity theory ratios themselves. With no other criterion to judge an interaction, the simplest rule to use for evaluation would be that of “giving to every man his own” (Hobbes, 1996, p. 96). However, the above-mentioned rule of interaction may not be the only one involved.

### Equity, Justice, and Agreement

Equity rules are but few of a broader set of possible distributional rules that are used to evaluate justice. Concerning the final distribution of outcomes, Deutsch (1975) argued that there are eleven different allocation rules that may lead to a fair outcome. These rules could be classified under three main categories: equity rules, equality rules, and need-based rules. Therefore, a result can be distributively fair if (a) outcomes are taken into account and found equal, (b) inputs are also taken into account and the ratios suggested by equity theory are found equal, or (c) needs are added in the equation and satisfied in a proportional manner. A different focus was introduced through Thibaut and Walker’s (1975, 1978) seminal work on the control that the disputants have on the legal procedure. Emphasis was placed on the fairness of the procedure and not necessarily that of the outcome and, thus, the concept of procedural justice was distinguished clearly from the concept of distributive justice. Another distinct concept is that of interactional justice (see Bies and Moag, 1986) that focuses on the relationship of the parties and not on the outcome or the procedure. More recently, Folger (2001) argued in favor of the concept of deontic justice since earlier conceptions “*ignore principled moral obligations and instead substitute personal desires as reasons for acting fairly or responding negatively to injustice*” (Cropanzano et al., 2003, p. 1019).

So many are the possible rules and norms that are used to evaluate justice that it seems legitimate to argue that any type of rule can serve as a criterion for evaluating justice. For example, disproportional outcomes may be judged fairer in the context of positive relational ties (Sherf and Venkataramani, 2015) or in case exchange participants are able to exercise autonomy in their task decisions (Cropanzano and Folger, 1989). At the same time, people may evaluate justice on the basis of the information that they receive first (Van den Bos et al., 1997). Cross-cultural considerations, such as individualism-collectivism, may also play an important role in justice evaluations (Chen et al., 1998).

Jasso (1980, 2005) goes beyond equity theory and offers a comprehensive justice analysis on the basis of a referential standard, the “just share,” which may be based on an aggregate set of comparisons, comparison with a group, or comparison with an abstract standard or principle. Hegtvedt and Johnson (2000) point out that equity theory assumes a single rule as legitimate without considering different aspects of legitimation. According to them, justice research should focus less on comparisons between the ratio of outcomes to inputs of a person and the ratio of another person; it should focus more on the person’s assessment of the fairness of that ratio and what other people assess as fair. However, their research does not necessarily reject the ‘instrumental rationality’ framework of classic equity theorists (see Hegtvedt et al., 2003).

Habermas (1985) offers a conception of rationality, namely communicative rationality, which is quite different to the self-interested instrumental rationality that equity theory accepts. In his work on morality (Habermas, 1991), he argues that ‘ought’ is established within reasoned discussion and by means of agreement. The evaluation of rules, or normative rightness in general, as well as the obligation to uphold rules can be approached on the basis of the rules’ potential validity in the context of reasoned discussion among members of an exchange relationship. It is useful to point out that empirical research can approach justice evaluations on the basis of agreement among participants in a discussion (Kahn et al., 1982). Heider (1958) also proposes that ‘ought’ is shared by all people in a particular situation, has intersubjective validity, and, moreover: “*people should concur in its directives*” (Heider, 1958, p. 222). This type of intersubjective validity often goes beyond the particular context, stands the test of a universal audience (Perelman and Olbrechts-Tyteca, 1958) and is supported by reasons that establish the rightness of norms during discourse (Habermas, 1991). Furthermore, every negotiation regarding an exchange can be viewed as an intersubjective process that validates the rights of negotiating parties (Arvanitis and Karampatzos, 2013), through the central role of agreement (Arvanitis, 2015). Therefore, inputs and outcomes do not necessarily determine the way in which justice is evaluated, since the evaluation is based on the rules that elicit agreement during discourse (rules that can go beyond equity).

The role of agreement has also been stressed in the realms of another social exchange theory, psychological contract theory (Rousseau, 1995). Psychological contract theory accepts that it is agreement that creates obligations to uphold certain norms, although people may fail to share the same understanding concerning the terms of the exchange (Dabos and Rousseau, 2004). In fact, psychological contract theory primarily investigates divergence of beliefs concerning the terms of interaction, studying, among others, how breach of a psychological contract is associated with feelings of violation (Robinson and Morrison, 2000). The appropriateness of a norm regarding an exchange is often established on the basis of a synthesis of different and contradicting perceptions. Habermas (1985, 1991) argues that such synthesis is accomplished by means of agreement during reasoned discussion.

### Attribution and Evaluation in Exchange: Two Distinct Cognitive Processes

Kelley and Michela (1980) made a crude distinction between attribution and “attributional” research. Attribution research examines or involves the manipulation of the antecedents of attributions, but is not interested in consequences beyond the attributions themselves. On the other hand, attributional research focuses more on the consequences of attribution in areas such as achievement or romantic love. What we find most interesting about this distinction is the attempt to dissociate the qualities of the attribution process itself from its consequences. This is exactly what we intend to do in the context of this research. Inputs and outcomes will initially be manipulated to understand how they contribute to attributions of outcomes and further evaluation will be treated as a separate cognitive process, an “attributional” process regarding the evaluation of fairness. Our research relies on minimal context in order to examine basic reasoning processes.

## Study 1: When Do People Perceive A Situation As Exchange?

The primary purpose of Study 1 is to examine whether people perceive each other’s influence as exchange, or more simply put, as give-and-take. More specifically, we will examine whether equity theory ratios accurately reflect the attribution of outcomes and provide the basis of perceptions of receiving in interdependent situations. We will further examine perceptions of justice to see how people evaluate the situation, since equity theory holds an eminent position in the justice literature. However, we will not include any aspect of communication between the hypothetical dyad of exchange participants.

Our general hypotheses concerning participants’ perceptions in a two-party interaction are derived from a simple analysis of **Figure 1**. Just by looking at the amount of inputs and outcomes of A and B and without further knowledge of their interaction (as well as the presence of any third factors) we would hypothesize that counterparts will be perceived either to have cooperated and proceeded with an exchange or to have proceeded individually on their own if inputs and outcomes were proportional. The case for cooperation would be more evident in the case that A and B contributed equally and had an equal share of the outcomes, because this type of situation would likely be the product of some sort of *intentional* exchange between A and B, instead of being some type of coincidence. The role of intentions in attribution processes has been repeatedly stressed in attribution theories, especially correspondent inference theory (Jones and Davis, 1965). Otherwise, a disproportional situation will lead to the perception (as long as a third factor is non-existent) that A has received from B or the opposite. Moreover, in the absence of any information concerning the normative rightness of potential rules, proportional situations will be perceived as fair, whereas disproportional situations as unfair.

Therefore, in proportional situations, counterparts would be perceived to receive equally from each other (Hypothesis 1A). As long as there is no other rule governing the interaction, the situation will be deemed just in cases of proportionality (Hypothesis 1B). In cases of disproportionality though, one person will be perceived to receive from the other in a way consistent with the direction and the size of the disproportionality (Hypothesis 2A). As long as there is no other rule governing the interaction, the situation will be deemed unjust in cases of disproportionality (Hypothesis 2B).

### Method

#### Participants and Design

Fifty four (18.52% males) undergraduate psychology students voluntarily participated in this study. Participants were between 18 and 25 years old with a mean age of 19.57 years old (*SD* = 1.07), all Caucasian. A 3 (balance of outcomes: balanced–*A* = *B*, imbalanced favorable to A–*A* > *B*, imbalanced favorable to B–*A* < *B*) × 3 (balance of inputs: balanced–*A* = *B*, imbalanced favorable to A–*A* < *B*, imbalanced favorable to B–*A* > *B*) within-subjects design was employed. The structure of the design (depicted in **Table 1**) was such that three of the resultant conditions exemplified proportionality (either balanced or imbalanced with reference to A or B) and six exemplified disproportionality (four reflected mild disproportionality due to imbalance in inputs or outcomes, and two reflected strong disproportionality).

**Table 1:** Design of study 1.

Outcomes A = B			Outcomes A > B			Outcomes A < B		
		
Inputs A = B	Inputs A > B	Inputs A < B	Inputs A = B	Inputs A > B	Inputs A < B	Inputs A = B	Inputs A > B	Inputs A < B
Balanced proportionality (BP)	Mild disproportionality (MDi) due to imbalance in inputs	Mild disproportionality (MDi) due to imbalance in inputs	Mild disproportionality (MDo) due to imbalance in outcomes	Imbalanced proportionality (IPa) with reference to A	Strong disproportionality (SD)	Mild disproportionality (MDo) due to imbalance in outcomes	Strong disproportionality (SD)	Imbalanced proportionality (IPb) with reference to B


#### Procedure and Materials

Participants were informed of the general purpose of the study and gave written consent, in line with the WMA declaration of Helsinki. No deception concerning the purpose of the study was necessary. As the stories used in this study involved the evaluation of hypothetical third-party interactions, we anticipated minimal emotional involvement on the part of participants. The procedure was approved by the supervisory committee of the Ph.D. thesis of the first author. After completion, participants were thoroughly debriefed and excused.

Participants were given a booklet containing nine short hypothetical scenarios. The basic scenario depicted A and B as business partners, and also presented their contribution to the company and their share of the profits. The scenario simply read “A and B are the sole partners in a company. The course of the company is entirely in their hands. The contribution of each to the company and the share of the profits of each are as follows:” Two bar graphs followed that showed A’s and B’s relative contribution to the company and their corresponding share of profits. The stories were minimal and were pilot tested in a small sample of university students to check comprehension and potential involvement. A short introductory page stressed that the information given would be limited, but encouraged participants to imagine such exchange situations and estimate the level of receiving for the hypothetical exchange dyad. Nine variations of the same story – which appeared randomly within a nine-page questionnaire – reflected the nine conditions of proportionality and disproportionality (see **Table 1**). Each scenario was followed by items designed to obtain measures for receiving (two short straightforward items that tapped into the perception of exchange abstractly: “*I think that A ‘received’ from B*,” “*I think that B ‘received’ from A*”) and one item to measure a general sense of justice of the situation (“*I think that the situation is just*”). Similar items were considered and dropped after the pilot study, after exhibiting very high correlations with the ones chosen, in an effort to minimize participant fatigue. Participants used a 7-point scale to indicate their agreement or disagreement with the items (7 for *strong agreement*, 1 for *strong disagreement*).

### Results

#### Receiving

We performed two separate analyses designed to test our hypotheses concerning the effects of proportionality (Hypothesis 1A) and disproportionality (Hypothesis 2A) on perceptions of receiving.

##### Proportionality

We performed a 3 (proportionality balance: balanced proportionality- BP, imbalanced proportionality with reference to A-IPa, imbalanced proportionality with reference to B-IPb) × 2 (receiving A vs. B: A’s receiving from B, B’s receiving from A) within-subjects ANOVA (**Table 2**). The main effect for ‘receiving A vs. B’ [*F*(1,53) < 1] and the two-way interaction were not significant [*F*(1,53) < 1], which indicates that there was no difference between A’s receiving and B’s receiving, a finding in support of Hypothesis 1A stating that in cases of proportionality counterparts would be perceived to receive equally from each other. However, there was a significant main effect of proportionality balance, *F*(2,106) = 35.30, *p* < 0.001, $\eta_{p}^{2}$ = 0.40; *post hoc* comparisons showed that perceptions of receiving were significantly higher for Balanced Proportionality (*M* = 4.98) than those for either condition of Imbalanced Proportionality (*M* = 3.54, *p* < 0.001; *M* = 3.53, *p* < 0.001). The difference between the latter IP conditions (IPa vs. IPb) was on average not significant. In other words, participants perceive that A and B receive from each other in BP whereas they do not have the same conviction in IP situations. In fact, one-sample *t*-tests revealed that the mean for BP (4.98) was significantly higher (*p* < 0.001) than the midpoint of the scale (4), indicating perceptions of receiving, while both means for IP (3.54; 3.53) were significantly lower (*p* < 0.05) than the midpoint of the scale (4), indicating that participants would tend to disagree that A or B received from each other. In other words, it can be argued that participants did not perceive the situation as one of exchange in the IP situation. The present findings allude to the fact that BP situations are perceived as exchanges whereas IP situations may not even be perceived as exchanges.

**Table 2:** Means (M) and standard deviations (SD) of receiving in conditions of proportionality.

	A’s receiving			B’s receiving		
		
	BP	IPa	IPb	BP	IPa	IPb
*M*	4.98	3.55	3.72	4.98	3.53	3.35
*SD*	1.13	1.12	1.34	1.27	1.10	1.36


*BP, balanced proportionality; IPa, imbalanced proportionality with reference to A; IPb, imbalanced proportionality with reference to B.*

##### Disproportionality

Given our Hypothesis 2A that disproportionality (i.e., conditions of either outcomes or inputs imbalance) lead to perceptions of unequal receiving from each other, reflecting the direction and the size of the disproportionality, it was thought most appropriate to calculate difference scores (measures of A’s receiving from B minus B’s receiving from A, for disproportionality favorable to A, and vice versa for disproportionality favorable to B) as more accurate reflections of who actually receives and to what extent. We performed a 3 (level of disproportionality: Strong Disproportionality-SD, Mild Disproportionality due to inputs-MDi, Mild Disproportionality due to outcomes-MDo) × 2 (Disproportionality favorable to: A or B) within-subjects ANOVA, on these difference scores (**Table 3**).

**Table 3:** Means (M) and standard deviations (SD) of difference scores of receiving in conditions of disproportionality.

	Disproportionality favorable to A			Disproportionality favorable to B		
		
	SD	MDi	MDo	SD	MDi	MDo
*M*	4.05	3.27	2.96	3.70	3.03	2.98
*SD*	2.26	2.14	2.30	2.43	2.45	2.61


*SD, strong disproportionality; MDi, mild disproportionality due to imbalance in inputs; MDo, mild disproportionality due to imbalance in outcomes.*

There was a main effect of level of disproportionality, *F*(2,106) = 7.08, *p* < 0.001, $\eta_{p}^{2}$ = 0.12; *post hoc* comparisons showed that perceptions of receiving were significantly higher for SD (*M* = 3.88) than those for either the condition of MD due to imbalance in inputs (*M* = 3.15, *p* < 0.001) or the condition of MD due to imbalance in outcomes (*M* = 2.97, *p* < 0.001)^1^. The difference between the latter two conditions was not significant. This finding supports Hypothesis 2A which states that in cases of disproportionality parties are perceived to receive in a way consistent with the direction and the size of the disproportionality since this perception is stronger in conditions of SD than in conditions of MD.

#### Justice of the Situation

We performed two separate analyses designed to test our hypotheses concerning the effects of proportionality (Hypothesis 1B) and disproportionality (Hypothesis 2B) on perceptions of justice.

##### Proportionality

A within-subjects one-way ANOVA (proportionality balance: balanced proportionality-BP, imbalanced proportionality with reference to A-IPa, imbalanced proportionality with reference to B-IPb) was performed (**Table 4**). The effect of level of proportionality on perceptions of justice was significant, *F*(2,106) = 13.40, *p* < 0.001, $\eta_{p}^{2}$ = 0.95. Perceptions of justice were significantly higher in the condition of BP (*p* < 0.001), than in either of the IP conditions, which did not differ significantly. One-sample *t*-tests revealed that all three means were significantly higher (*p* < 0.001) than the midpoint of the scale (4) indicating strong perceptions of justice. These findings are in line with Hypothesis 1B stating that in cases of proportionality the situation will be deemed just; however, in BP the situation is deemed more just than in IP.

**Table 4:** Means (M) and standard deviations (SD) of justice in conditions of proportionality.

	BP	IPa	IPb
*M*	6.51	5.55	5.44
*SD*	0.66	1.90	1.95


*BP, balanced proportionality; IPa, imbalanced proportionality with reference to A; IPb, imbalanced proportionality with reference to B.*

##### Disproportionality

We performed a 3 (level of disproportionality: Strong Disproportionality-SD, Mild Disproportionality due to inputs-MDi, Mild Disproportionality due to outcomes-MDo) × 2 (Disproportionality favorable to: A or B) within-subjects ANOVA, on perceptions of justice (**Table 5**). There was only a significant main effect of level of disproportionality, *F*(2,106) = 10.00, *p* < 0.001, $\eta_{p}^{2}$ = 0.34; *post hoc* comparisons showed that perceptions of justice were significantly higher for MD due to imbalance in inputs (*M* = 2.13) than those for either the condition of MD due to imbalance in outcomes (*M* = 1.84, *p* < 0.05) or the condition of SD (*M* = 1.60, *p* < 0.001). The difference between the latter two conditions was not significant. The theoretical explanation for this finding is that in cases of disproportionality, equality in outcomes (as is the case in MDi) renders the situation less unjust than other disproportional situations. In all cases of disproportionality, however, the situation was deemed unjust, since one-sample *t*-tests revealed that all three means were significantly lower (*p* < 0.001) than the midpoint of the scale (4), thus indicating perceptions of injustice, a finding consistent with Hypothesis 2B.

**Table 5:** Means (M) and standard deviations (SD) of justice in conditions of disproportionality.

	Disproportionality favorable to A			Disproportionality favorable to B		
		
	SD	MDi	MDo	SD	MDi	MDo
*M*	1.53	2.05	1.83	1.66	2.20	1.85
*SD*	0.77	1.13	1.12	1.22	1.23	1.03


*SD, strong disproportionality; MDi, mild disproportionality due to imbalance in inputs; MDo, mild disproportionality due to imbalance in outcomes.*

### Discussion

In study 1 we examined the effect of inputs and outcomes on perceptions of receiving and justice in order to test our hypotheses, which were formulated along the lines of equity theory. Our main rationale was that equity theory ratios would serve as the basis of perceptions of receiving – thereby revealing the underlying attribution process of outcomes to inputs–, as well as influence perceptions of justice in the absence of any other information regarding the communication between the hypothetical actors.

In conditions of balanced *proportionality* (i.e., inputs and outcomes are equal) the perceptions of receiving and justice are high. In conditions of imbalanced proportionality (imbalance both in inputs and outcomes that cancels out), it seems as if it were “every man for himself.” No one is really perceived to receive from the other and this absence of influence is deemed fair. We can argue that a proportional interdependent situation will be perceived as exchange when it is balanced, probably because balanced situations require coordination and some type of exchange in order to be achieved. Otherwise, the situation might not even be perceived as exchange, as outcomes will not be attributed to inputs of other individuals. Moreover, and as we expected on the basis of equity theory, proportional situations are generally perceived as fair. However, it is notable that perceptions of justice are higher when exchange is perceived to have taken place, as exemplified in the balanced proportionality condition.

The results for *disproportionality* are a little less straight forward. There is definitely a sense of receiving in dispro portionality and it gets more intense as the disproportionality gets stronger. This is consistent with expectations based on equity ratios (Hypothesis 2A). Moreover, it is definitely perceived as unjust for one party to receive more than the other (thereby supporting Hypothesis 2B). However, perceptions of justice do not follow the same pattern as that detected for receiving since stronger disproportionality did not lead to higher perceptions of injustice, which means that the level of perceived injustice is not based adequately on equity theory ratios. In this case, the most plausible explanation is that equality (i.e., balance in outcomes) rather than equity was more salient in participants’ responses concerning the level of injustice in conditions of disproportionality.

We have demonstrated that equity ratios are consistent with perceptions of who receives more in a relationship; they appear to serve as causal schemas, as indicated by the overall support of our hypotheses. Judgment or evaluation of the exchange, as a separate cognitive process, could on the other hand be the result of the application of any rule or norm (in this case it involved both an equity and an equality rule). The examination of this issue is one of the primary aims of study 2.

## Study 2: Are Inputs and Outcomes the Only Factor in the Evaluation Process?

The purpose of study 2 is twofold: To replicate the findings of study 1 concerning disproportionality across three types of interaction situations and further to examine if equity ratios serve more as causal schemas of exchange than instruments of justice evaluations. Our goal is to show that apart from the cognitive process of attribution of outcomes, there is another cognitive process that is responsible for the evaluation of the situation as just, one that might not be captured at all by equity theory. We propose that in cases of disproportionality, although the favored person is perceived as having received, this is not necessarily seen as unjust. We have already argued that the evaluation process might involve the application of a rule or norm that is agreed upon during reasoned communication. Thus, a disproportional situation might be seen as just if there is a communicatively rational rule pointing in that direction or, more particularly, a reason for this disproportionality that is validated by the agreement of the parties involved (a rule that is *reciprocally* accepted by parties). More specifically, we hypothesize that: In disproportional situations, irrespective of input-outcome structure or the existence of agreement, the favored party is perceived as having received (Hypothesis 3) but, disproportionality will not be seen as unjust if it is rationally agreed upon through reasoned communication (Hypothesis 4).

### Method

#### Participants and Design

Sixty undergraduate psychology students voluntarily participated in this study. The sample consisted of 16.6% males and 83.3% females. Participants were between 18 and 38 years old with a mean age of 20 years old (*SD* = 2.96), while all of them were Caucasian. A 3 (games: chicken, prisoners’ dilemma, assurance) × 2 (communication: no-agreement, agreement) within-subjects design was used.

#### Procedure and Materials

Participants gave written consent after being informed of the general purpose of the study, in line with the WMA declaration of Helsinki. As in Study 1, no deception was employed and minimal emotional involvement on the part of participants was expected. The procedure was approved by the supervisory committee of the Ph.D. thesis of the first author.

Participants were given a booklet containing three versions of the story of A and B who are business partners, a story quite similar to the one used in study 1. The three versions of the story (variations involved the existence of a bonus or not, the amount of work, the level of pay) reflected the utility structure of three types of games, very common in game theory research literature – chicken, prisoner’s dilemma, assurance (Poundstone, 1992). The prisoner’s dilemma concerns two prisoners offered a Faustian bargain by the police to testify against each other and go free. Game theory predicts that rational pursuit of self-interest would result in both prisoners backstabbing each other (i.e., “defecting” instead of “cooperating”). Chicken, otherwise known as hawk-dove, is modeled after the teenage game of highway chicken where two drivers set their vehicles on collision course and the first one who swerves, loses. In this case, the rational solution is not where both players defect, for that outcome will result in their death; the rational solution is where one player swerves (or “cooperates”) and the other drives straight (or “defects”). Assurance, otherwise known as stag hunt, trust dilemma or cooperation game, is not a real dilemma at all. All parties prefer to cooperate and hunt a stag but the possibility that someone might defect does not make catching the stag a certainty. In this case, lack of trust or miscommunication might make someone hunt a hare (i.e., “defect”) than hunt the stag (i.e., “cooperate”).

These games were chosen to illustrate different input-outcome structures, which were presented by emphasizing A’s and B’s structure of preferences (see Appendix), and to test whether different strategies within each scenario would influence perceptions of receiving or justice. The three games were presented to participants in the aforementioned order. In both prisoner’s dilemma and chicken, A and B (the characters in the story) have an interest in deviating from a situation in which they both work a lot (i.e., the situation in which they “cooperate”). Only in the assurance game, the situation in which they both work a lot is one they would not want to deviate from. After receiving information on each version of the story, participants were told that the eventual outcome of the story was a disproportional one (favorable to B) in all three cases. Each version was followed by items designed to obtain measures for receiving (“*I think that B ‘received’ from A*”) and justice (“*I think that the situation is unjust*”). Participants used a 7-point scale to indicate their agreement or disagreement with the items (7 for *strong agreement*, 1 for *strong disagreement*). Participants responded to the same items under two conditions: Under the premise that there is agreement that allowed for the disproportionality for reasons regarding the personal relationship of A and B (agreement condition) and under no such premise (no-agreement condition). The agreement condition followed the no-agreement condition – which essentially served as the control – for each of the three games. It should be stressed that in the agreement condition the story simply stated that the outcome was due to “their agreement, supported by reasons that had to do with their personal relationship” *without mentioning what those reasons could have been*. In this way we made agreement salient without mentioning what it might have entailed, although noting that it was supported in the context of their interaction^2^.

### Results

Given the directional nature of the hypotheses, the relevant tests are presented first, followed by further analysis using a three (games: chicken, prisoners’ dilemma, assurance) × 2 (communication: no-agreement, agreement) within subjects ANOVA (**Table 6**).

**Table 6:** Means (M) and standard deviations (SD) of B’s receiving and injustice.

		No-agreement			Agreement		
			
		Chicken	PD	Assurance	Chicken	PD	Assurance
B’s receiving	*M*	5.30	5.38	4.78	4.75	4.90	4.73
	*SD*	1.13	1.12	1.34	1.27	1.10	1.36
Injustice	*M*	5.23	5.25	4.66	3.41	3.25	3.38
	*SD*	1.37	1.43	1.42	1.48	1.43	1.51


#### Receiving

One-sample *t*-tests revealed that, in all six conditions, means for perceptions of B’s receiving were significantly higher (*p* < 0.001) than the midpoint of the scale (4), indicating that participants in all conditions perceived that the favored party B did receive from A. These findings are consistent with Hypothesis 3. The ANOVA on perceptions of B’s receiving revealed a significant main effect of games, *F*(2,118) = 5.98, *p* < 0.01, $\eta_{p}^{2}$ = 0.09 and a significant main effect of communication, *F*(1,59) = 8.11, *p* < 0.01, $\eta_{p}^{2}$ = 0.12. However, these main effects were qualified by a significant games × communication interaction, *F*(2,118) = 4.93, *p* < 0.01, $\eta_{p}^{2}$ = 0.08. The breakdown of this interaction revealed the following: In the no-agreement condition, perceptions of B’s receiving in the assurance game condition (*M* = 4.78) were significantly lower than those in both other conditions (*M* = 5.38, *p* < 0.001 for prisoners’ dilemma; *M* = 5.30, *p* < 0.01 for chicken), while the latter two conditions did not differ significantly; this game effect was not significant in the agreement condition. It appears that perceptions of B’s receiving did not differ across games when there was agreement on the disproportionality, while they were higher in the prisoners’ dilemma and chicken conditions when no agreement was mentioned. On the whole, these findings lend further support to the findings of study 1 concerning perceptions of receiving in cases of disproportionality.

#### Injustice

One-sample *t*-tests revealed that, in the agreement condition, means for perceptions of injustice for all three games were significantly lower (*p* < 0.001) than the midpoint of the scale (4), indicating low perceptions of injustice. These results are consistent with Hypothesis 4. The ANOVA on perceptions of injustice revealed a significant main effect of games, *F*(2,118) = 3.24, *p* < 0.05, $\eta_{p}^{2}$ = 0.05 and a significant main effect of communication, *F*(1,59) = 72.99, *p* < 0.001, $\eta_{p}^{2}$ = 0.55 showing that perceptions of injustice in the no-agreement condition (*M* = 5.05) were significantly higher than in the agreement condition (*M* = 3.35). However, these main effects were qualified by a significant games × communication interaction, *F*(2,118) = 6.69, *p* < 0.01, $\eta_{p}^{2}$ = 0.10. The breakdown of this interaction revealed the following: in the no-agreement condition, perceptions of injustice for the assurance game (*M* = 4.66) were significantly lower than those in both conditions of prisoners’ dilemma (*M* = 5.25, *p* < 0.01) and chicken (*M* = 5.23, *p* < 0.01), while the latter two conditions did not differ significantly. In the agreement condition this game effect was not significant.

To sum up, it appears that perceptions of injustice differ across the communication conditions. Once there is agreement (a reciprocally accepted rule) on the disproportionality, the situation is no longer seen as unjust (although it is perceived that B still receives from A, as we saw in the analysis of receiving). It is worth noting, however, that in the no-agreement condition, perceptions of injustice are lower in the assurance game (which is consistent with the perception that B receives less in the assurance game), a finding which will be further discussed.

### Discussion

In study 2 we examined participants’ perceptions of receiving and injustice for a disproportional outcome in an interdependent situation across three types of games and under two conditions of agreement and no-agreement on this disproportionality. *In the case of the existence of agreement* within the relationship, disproportionality was not seen as unjust across all three types of games. This runs counter to equity-based expectations concerning perceptions of justice. At the same time, perceptions of receiving were in line with equity-based expectations about attributions and were the same across all three types of games. In other words, it can be argued that individuals use equity ratios to attribute outcomes and understand patterns of receiving but use norms and terms that evoke rational agreement in order to understand justice.

Although our purpose was to distinguish between agreement (as the only factor affecting perceptions of justice) and the structure of inputs-outcomes (as the only factor affecting perceptions of receiving), we have to acknowledge that there is a relationship between perceptions of receiving and perceptions of agreement, as operationalized, measured and tested in the above study. The interaction between agreement and input-outcome structures shows that agreement can be treated as the main objective of communicative rationality mechanisms (as we intended) but can also be treated as something you give or take from other people, just like a promise or a favor or indeed, any type of resource. This would account for the finding that, where explicit agreement was absent (no agreement condition), perceptions of receiving were higher in games (Prisoners’ Dilemma and Chicken Game) where hypothetical actors appeared to consciously deviate from implicit agreements of “cooperation”–thus receiving more from their exchange counterparts-. In the assurance game though, where each of the hypothetical actors had no way of knowing what the other would do and thus there was no hint of implicit agreement that was “violated,” perceptions of receiving were lower. This is useful to point out since treating agreement in this way is equivalent to considering agreement as another type of resource that weighs in with regard to attribution processes rather than treating it as the ultimate criterion for the appropriateness of norms and justice considerations.

Despite small differences in perceptions of receiving, *disproportionality does create perceptions of receiving across all types of games and conditions of agreement*. From then on, perceptions of justice may be based on the usual type of rules: that of “giving to every man his own” (Hobbes, 1996, p. 96). This is the exact pattern that we saw in the no-agreement condition. On the other hand, perceptions of justice can be based on reasoning such as “Elderly people should receive more” or “Women should be paid less” (Moore, 2006) or, indeed, on any rules that can be supported in reasoned discussion, even if they point toward disproportionality and inequity. Equity theory alone cannot account for perceptions of justice – and consequently, for the findings in the agreement condition. It is evident that in study 2, *an explicitly reciprocally accepted rule influenced perceptions of justice substantially*. Moreover, these basic conclusions seem to generalize across all types of interactions (illustrated in this study by the different types of games). This means that independent of the utility structure of any interaction, people will perceive the direction of the give-and-take on the basis of the attribution of outcomes but will judge what is just or not, according to the potential *communicatively rational* agreement on the rules they see relevant. The present findings seem to provide initial support for our contention that there are two distinct cognitive processes in social exchange: one concerning the attribution of outcomes and another pertaining to the evaluation of the situation.

## General Discussion

Social exchange theory can study how people affect each other by the use of their resources, which can broadly be defined as behavioral capabilities (Emerson, 1981), and in this way explain a wealth of social psychological phenomena, especially in the organizational context where behavior is often treated as part of an exchange process. In this paper we have demonstrated that situations of interdependence can often be perceived as give-and-take situations. On the basis of a causal schema that treats inputs as causes and outcomes as effects, we proposed that people attribute outcomes of a social interaction either to their own or to other people’s inputs and further argued that, when they attribute outcomes to the inputs of other people, they treat an interaction as exchange. Not all situations are necessarily treated as exchange though. Our findings indicate that an imbalanced proportionality situation, that is, a situation where the equity ratios are equal without inputs or outcomes being equal at the same time, it is “every man for himself”: People do not seem to attribute their own outcomes to the inputs of other people. On the other hand, situations of disproportionality or balanced proportionality create strong perceptions of receiving from one another.

Equity ratios therefore represent a causal schema that appears rudimentary in an ‘interaction as exchange’ situation. This type of approach offers great potential in understanding how people attribute outcomes to possible underlying exchanges. Let’s for example take the outcome of John’s high employee performance: Attributing the performance to his own good skills may weaken the perception of John receiving from Jane, his fellow employee. On the other hand, in a closed system of a two employees’ unit, an assumption that John has low skills leads to the attribution of the ‘high performance’ outcome to inputs from Jane, which in turn implicitly points to an underlying exchange on the basis of the disproportionality of the situation. At the same time, a multiple necessary input-outcome schema (see Kelley, 1972) can lead to the conclusion that John both has good skills *and* has received from fellow employees. If both John and Jane have high performance and high skills and on the basis of our research findings, we will predict that the business interaction between John and Jane will be perceived as exchange, since it is a balanced proportional situation. On the other hand, if John has low performance and low skills, whereas Jane has high performance and high skills, the situation will most likely not be perceived as exchange. Indeed equity ratios can serve as a causal schema that can be applied in many social exchange situations and in many different ways that can be predicted by attribution theory.

Inputs and outcomes by themselves cannot determine how people will judge the situation once they perceive it as exchange, contrary to what equity theory might suggest. On the basis of our findings it seems legitimate to suggest that people are able to make judgments through rules, norms, values, terms that can be supported within the context of reasoned communication leading to agreement. Although not all real-life agreements necessarily validate the rightness of norms and especially those that determine justice evaluations, it was shown that agreement can serve as a strong criterion for evaluating a situation as just, even in the absence of any other information concerning an interaction. This finding underlines the importance people place on agreement among individuals in evaluating the fairness of any situation. By accepting the broader conception of communicative rationality and contrasting it with instrumental rationality (Habermas, 1985), we can approach exchange situations and the different criteria (distributive, procedural, interactional, deontic) for evaluating the situation by focusing on a cognitive process that essentially tests the validity of any norm through the examination of what would be reasonable during communication. In the previous example of John and Jane, any type of arrangement will be judged according to what would appear reasonable in rational discussion and in the end, according to what would elicit agreement. Therefore proportional outcomes might be judged as unfair or disproportional outcomes as fair, according to communicative rationality mechanisms.

The two studies presented can be seen as a first attempt in assessing equity ratios as causal schemas and as such they have some limitations. Firstly, we only used a ‘closed’ system of two actors in which no other factors really played a role in the outcome of the exchange. We excluded third factors that might be incorporated through the presence of other actors or environmental factors such as chance. Needless to say that a factor such as chance (for chance attributions see Rotter et al., 1962; cf. Friedland, 1992) would arguably make the attribution of outcomes to the inputs of other people less probable and consequently the perception that exchange took place weaker. Secondly, we used a third-person perspective in order to examine pure cognitive processes that were as basic as possible, but it would be interesting to examine these processes from the viewpoint of participants in more ‘open’ exchange situations, either in the laboratory or in real life, especially when they are actively involved. It has been shown that people perform justice evaluations in a self-serving manner (De Dreu, 1996) or that taking the perspective of other exchange participants reduces enjoyment for high credit claimers (Epley et al., 2006). Reciprocation in kind is also subject to egocentric interpretations of the exchange process (Zhang and Epley, 2009). It would be useful to further examine how instrumentally rational considerations influence communicatively rational perceptions since, arguably, any type of judgment is often put to the test of a dialectic process, even if it is self-interested (see Arvanitis and Karampatzos, 2011). Finally, we examined the use of positive resources, but the study of negative resources would be equally important.

In this paper, we have attempted to distinguish between the cognitive processes of attribution and evaluation in an exchange situation. We have generally supported our general contention that attribution and evaluation can be distinguished as different cognitive processes. Further exploration as to how these two cognitive processes are exactly linked could be potentially useful in understanding more about other processes such as prescriptive attributions (Gollan and Witte, 2008) and in offering links among social exchange theory, equity theory, psychological contract theory and communicative rationality mechanisms.

## Author Contributions

All authors listed, have made substantial, direct and intellectual contribution to the work, and approved it for publication.

## Conflict of Interest Statement

The authors declare that the research was conducted in the absence of any commercial or financial relationships that could be construed as a potential conflict of interest.

## Footnotes

1 Since we are dealing with difference scores based on 7-point scales (min = 0, max = 6), the higher the score the stronger the perception that one party receives.

2 It should be stressed that this experiment does not really compare distributive, procedural, or interactional justice rules, but rather examines whether the existence of any type of rule (even if it is never revealed) could serve as an explanation of a seemingly unjust outcome (for example, by equity theory standards) once it is *mutually* accepted by parties.
