# Supplementary material for: Equity Theory Ratios as Causal Schemas
Source: Front Psychol. 2016 Aug 19;7:1257. doi: 10.3389/fpsyg.2016.01257 (PMC4990720; doi:10.3389/fpsyg.2016.01257)
Supplement: Supplementary file 1 [file Data_Sheet_1.DOCX]

**APPENDIX**

Below is the brief recapitulation of the stories as they were presented in the booklet given to participants in Study 2. Under each possible scenario, the preference structure was given in brackets in the way that is depicted below [e.g. (A1, B3) implies that the scenario would be the 1^st^ choice for A and the 3^rd^ for B]

| **CHICKEN** | | | | | | | |
| --- | --- | --- | --- | --- | --- | --- | --- |
| *A’s choice* | Hard Work | | |  | Light Work | | |
| *B’s choice* | Hard Work |  | Light Work |  | Hard Work |  | Light Work |
|  | The project succeeds, they each get a bonus but it takes up too much effort |  | Neither gets a bonus. A prefers B had worked enough so that they could have gotten the bonus. For B this is the most desirable scenario, since he didn’t put too much effort in the project |  | Neither gets a bonus. B prefers A had worked enough so that they could have gotten the bonus. For A this is the most desirable scenario, since he didn’t put too much effort in the project |  | The project fails. They do not get any pay. This is the least desirable option both for A and B |
| *Preferences* | (A2, B2) |  | (A3, B1) |  | (A1, B3) |  | (A4, B4) |

| **PRISONERS’ DILEMMA** | | | | | | | |
| --- | --- | --- | --- | --- | --- | --- | --- |
| *A’s choice* | Hard Work | | |  | Light Work | | |
| *B’s choice* | Hard Work |  | Light Work |  | Hard Work |  | Light Work |
|  | The project succeeds and both enjoy high earnings |  | The project succeeds and both enjoy high earnings. For B this is the most desirable scenario since he didn’t put too much effort in the project. For A this is the least desirable scenario since he put in a lot of effort. |  | The project succeeds and both enjoy high earnings. For A this is the most desirable scenario since he didn’t put too much effort in the project. For B this is the least desirable scenario since he put in a lot of effort. |  | The project partly succeeds and both enjoy slightly lower earnings. |
| *Preferences* | (A2, B2) |  | (A4, B1) |  | (A1, B4) |  | (A3, B3) |

| **ASSURANCE GAME** | | | | | | | |
| --- | --- | --- | --- | --- | --- | --- | --- |
| *A’s choice* | Hard Work | | |  | Light Work | | |
| *B’s choice* | Hard Work |  | Light Work |  | Hard Work |  | Light Work |
|  | The project succeeds and both enjoy high earnings |  | The project succeeds with moderate earnings. This is not really painful to B because he worked less. However, he would have preferred that they both had worked hard (there was just no way of knowing that A would have done so). For A this the least desirable scenario |  | The project succeeds with moderate earnings. This is not really painful to A because he worked less. However, he would have preferred that they both had worked hard (there was just no way of knowing that B would have done so). For B this the least desirable scenario |  | The project succeeds but the earnings are quite low. |
| *Preferences* | (A1, B1) |  | (A4, B2) |  | (A2, B4) |  | (A3, B3) |
